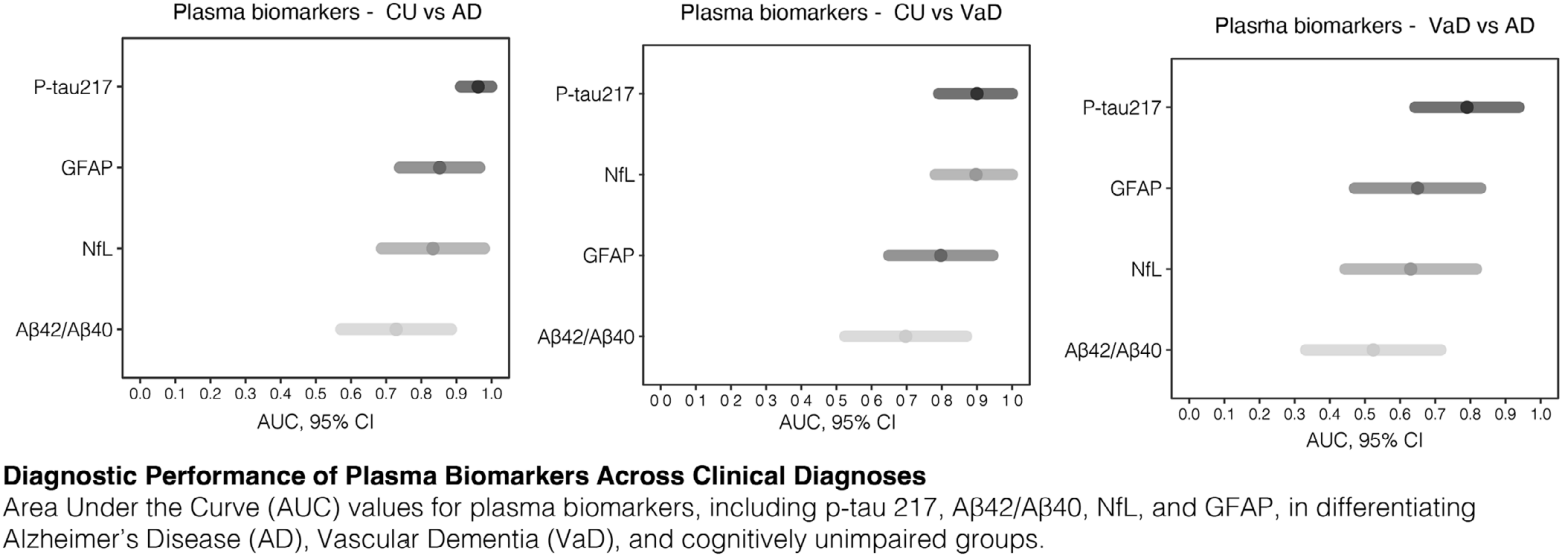# Alzheimer's Disease Plasma Biomarkers Performance in Brazilian Individuals

**DOI:** 10.1002/alz70856_099080

**Published:** 2025-12-24

**Authors:** Eduardo R. Zimmer, Wyllians Vendramini Borelli, Pamela C.L. Ferreira, Wagner S. Brum, João Pedro Ferrari‐Souza, Maila Rossato Holz, Victória Tizeli Souza, Matheus Zschornack Strelow, Carolina Rodrigues Formoso, Marcia L. Chaves, Giovanna Carello‐Collar, Andreia Silva da Rocha, Cristiano Aguzzoli, Guilherme Povala, Bruna Bellaver, Pedro Rosa‐Neto, Raphael Machado Castilhos, Tharick A Pascoal

**Affiliations:** ^1^ Universidade Federal do Rio Grande do Sul, Porto Alegre, RS, Brazil; ^2^ McGill University, Montreal, QC, Canada; ^3^ Brain Institute of Rio Grande do Sul ‐ Pontifícia Universidade Católica do Rio Grande do Sul, Porto Alegre, Rio Grande do Sul, Brazil; ^4^ Universidade Federal do Rio Grande do Sul, Porto Alegre, Rio Grande do Sul, Brazil; ^5^ University of Pittsburgh, Pittsburgh, PA, USA; ^6^ PUCRS, Porto Alegre, Rio Grande do Sul, Brazil; ^7^ Hospital de Clínicas de Porto Alegre, Porto Alegre, Rio Grande do Sul, Brazil; ^8^ Hospital de Clínicas de Porto Alegre, Porto Alegre, Brazil; ^9^ Hospital de Clinicas de Porto Alegre, Porto Alegre, RS, Brazil; ^10^ Brain Institute of Rio Grande do Sul (InsCer), Porto Alegre, Rio Grande do Sul, Brazil; ^11^ Departments of Psychiatry and Neurology, University of Pittsburgh School of Medicine, Pittsburgh, PA, USA

## Abstract

Alzheimer's disease (AD) biomarker research has largely concentrated on populations from the Global North. The emergence of blood‐based biomarkers presents an opportunity to reduce this disparity. In this perspective presentation, I will present data on the performance of blood‐based biomarkers in a real‐world, memory clinic‐based cohort from Brazil, a population characterized by lower educational attainment compared to those typically studied in the Global North. Specifically, I will examine the performance of plasma biomarkers—Aβ40, Aβ42, *p*‐tau217, NfL, and GFAP—in differentiating AD from cognitively unimpaired (CU) individuals and vascular dementia (VaD) in a Brazilian cohort (*n* = 59). Preliminary findings indicate that *p*‐tau217 exhibits the highest accuracy in distinguishing AD from CU (AUC 0.96). However, the performance of all plasma biomarkers in differentiating AD from VaD is lower (AUC 0.52 to 0.79) than expected based on studies conducted in the Global North. Finally, I will share initial findings from the Brazilian Initiative of Blood Biomarkers in Neurodegenerative Disorders, a program funded by the Ministry of Health. Additionally, I will discuss the role of blood biomarkers in shaping state and national dementia plans in Brazil.